# Analyzing One Cell at a TIME: Analysis of Myeloid Cell Contributions in the Tumor Immune Microenvironment

**DOI:** 10.3389/fimmu.2020.01842

**Published:** 2020-09-02

**Authors:** Vitaliy Davidov, Garrett Jensen, Sunny Mai, Shu-Hsia Chen, Ping-Ying Pan

**Affiliations:** ^1^Texas A&M College of Medicine, Bryan, TX, United States; ^2^Center for Immunotherapy Research, Cancer Center of Excellence, Houston Methodist Research Institute, Houston, TX, United States

**Keywords:** MDSC, TAM, TIME, sc-RNAseq, reprogram

## Abstract

Tumor-mediated regulation of the host immune system involves an intricate signaling network that results in the tumor's inherent survival benefit. Myeloid cells are central in orchestrating the mechanisms by which tumors escape immune detection and continue their proliferative programming. Myeloid cell activation has historically been classified using a dichotomous system of classical (M1-like) and alternative (M2-like) states, defining general pro- and anti-inflammatory functions, respectively. Explosions in bioinformatics analyses have rapidly expanded the definitions of myeloid cell pro- and anti-inflammatory states with different combinations of tissue- and disease-specific phenotypic and functional markers. These new definitions have allowed researchers to target specific subsets of disease-propagating myeloid cells in order to modify or arrest the natural progression of the associated disease, especially in the context of tumor-immune interactions. Here, we discuss the myeloid cell contribution to solid tumor initiation and maintenance, and strategies to reprogram their phenotypic and functional fate, thereby disabling the network that benefits tumor survival.

## Introduction

In recent decades the traditional view of tumor development and metastasis has evolved to include new and emerging cell types, extrinsic to the tumor itself. Over time it has become apparent that tumors are composed of many cell types from different origins, all with varying functions. By defining the tumor as a distinct organ, cell populations can be broadly separated into two categories: parenchymal tumor cells and stromal tumor-associated cells. Tumor-associated cells can originate either from the tissue in which the malignancy arises, or they can migrate from the periphery and infiltrate the tumor after it forms. The tumor itself and the tumor-associated cells together comprise what is termed the tumor microenvironment (1). When the tumor microenvironment being discussed relates to the influx and function of the immune system, it is termed the tumor immune microenvironment (TIME) (2). Therapies targeting different components of the tumor microenvironment, such as neovascularization, cellular proliferation, growth factors, extracellular matrix proteins, and more, have all been utilized to regulate tumor growth, each with various levels of success (3). More recently, targeting the immune component of the malignancy, deemed immunotherapy, has shown great promise and curative potential in several tumors (4).

Fundamentally, the goal of immunotherapy is to modulate the mechanisms that tumors use to suppress the immune response. The ability of a tumor to evade immune mediated killing is one of the hallmarks of cancer development, highlighting the importance of the immune response in preventing cancer formation (1). Classically, the immune system is divided into two branches: adaptive and innate. The innate division determines how to respond to danger by sensing the environment with an array of pattern recognition receptors and cytokine receptors that allow them to sense tissue damage, pathogens, and inflammation. The defining feature of the adaptive branch is its ability to respond in an antigen specific manner and memory responses (5). The importance of the innate immune system in regulating malignancies has come into sharper focus with the discovery of immunomodulatory myeloid cells residing within and around tumors. These myeloid cells are known to play a central role in suppressing adaptive immunity and are comprised of diverse clusters that fulfill various roles in promoting the viability of the developing malignancy. Two central groups of suppressive myeloid cells are the tumor-associated macrophages (TAMs) and myeloid-derived suppressor cells (MDSCs) (6). Initially called natural suppressor cells, these cells were shown to inhibit cytotoxic lymphocyte activity and support tumor growth (7). A body of work has shown that tumor development frequently causes defects in the differentiation and activity of myeloid cells, ultimately leading to a functional state that favors the tumor progression. Given the massive heterogeneity of infiltrating leukocytes found in tumors, and the striking difference in the TIME seen between different tumor types, there is a need to better understand the mechanisms contributing to this overall immune suppressive environment at the single cell and high-dimensional level. Advances in single-cell RNA sequencing (scRNAseq) and mass cytometry have enabled these types of studies and comparisons and are giving rise to new generations of data that may provide greater understanding of the mechanisms leading to immune suppression, TAM and MDSC polarization, and immune evasion.

Studies testing the potential of modulating the TIME via altering cellular recruitment, differentiation, proliferation, and survival are currently underway. These are reviewed elsewhere (8–11). Here, we discuss tumor associated suppressive myeloid cells, analyze recent findings obtained through high resolution dissection of their phenotypes, and highlight potential reprogramming strategies to orient cells toward anti-tumor functionality.

## The Players: TAMs and MDSCs

In the 1960's, it was first observed that tumor bearing mice developed a leukemoid reaction with expanded myeloid cell populations in both the circulation and in the tumor. This correlated with enhanced tumor growth and these cells were subsequently shown to suppress cytotoxic T cell activity (7). Over time additional research has demonstrated that these myeloid cells exist as two separate populations: TAMs and MDSCs (12). Studies seeking to understand the factors that led to the differentiation of these populations demonstrated that tumor-associated macrophages develop from both tissue resident and circulating monocyte populations (13). New myeloid cells recruited from the bone marrow exhibit different programming from embryonically derived tissue resident macrophages (TRMs) (14), and commonly represent the definition of “tumor-associated macrophage” populations (15, 16), albeit not without debate, depending on tumor model (17–19).

### Myeloid-Derived Suppressor Cells

Correctly identifying MDSCs *in vivo* remains challenging despite decades of intense study. MDSCs are commonly identified in tumor bearing mice by the Gr-1 surface marker, and recently, CD84 has arrived into the spotlight as another potential marker in murine models. There is potential for application of CD84 to differentiate MDSCs from conventional myeloid cells in human studies, but this has yet to be validated (20). Despite shortcomings in MDSC phenotypic definitions, several surface markers are employed in the literature with varying degrees of success and have been discussed elsewhere (12, 21). Thus, the gold standard and only reliable method to correctly identify MDSCs is to evaluate their ability to suppress CD3-mediated T cell activation and function *in vitro* (22–24).

MDSC recruitment and maintenance within the tumor tissue is thought to be more complex than that for TAMs, in part because of the hypothesized signaling required to maintain MDSCs in an immature state. This is thought to be accomplished by a combination of multiple growth factors and polyunsaturated fatty acids (25). Supplementary inflammatory signals generated by the tumor traps these immature cells in a pathogenic suppressive state (25, 26). A combination of TLR4/IFNγ/GM-CSF signaling and activation of intracellular STAT3 is needed to control the development and function of MDSCs (27–30). MDSCs are typically replenished by bone marrow precursors and the spleen functions as their reservoir (31), but it is unclear as to how extramedullary hematopoiesis contributes to their replenishment.

A growing body of evidence supports that MDSCs retain some ability to polarize to a cell displaying more characteristics of typical monocytes (32, 33). Genetic and pharmacologic methods can promote maturation or polarization in MDSCs, with multiple groups reporting that M-MDSCs can be functionally characterized into not only suppressive states, but also into reactive states (32, 34, 35). Transcriptional programs initiated by c-EBPβ, STAT3, PU.1, IRF8, and RORC1, among others, regulate the suppressive activities of MDSCs (36). Blocking these programs to force MDSCs into an activating, rather than suppressive role, is a potential therapeutic strategy, with several mechanisms to do so (37). MDSCs represent just one of the suppressive populations in the TIME; quantifying the phenotypes and functional states of the environment at the single cell level will offer more clues for therapeutic applications.

### Tumor-Associated Macrophage

Tumor-associated macrophages comprise the macrophage populations located in and around a solid tumor (38). Originating from both tissue resident macrophages and circulating monocytes, TAMs are also known to perform a prominent role in modulating immune responses to tumors (39). TAMs can arise from peripheral monocytes in response to a combination of CCL2 and CSF1 produced by the tumor (27–30, 40, 41). Once monocytes reach the tumor site, they follow a maturation course that leads to their TAM finale (42, 43), under the influence of tumor factors, local cytokine milieu, and integrin signaling (44). Other than replenishment of TAM populations, the role of undifferentiated monocytes within the TIME has not been clearly defined at the single-cell level. Additional important signaling pathways resulting in macrophage recruitment and subsequent TAM differentiation include VEGF, IL-4, CCL2, CCL18, and CCL9 (45). TAMs further mobilize additional TAMs to the tumor niche by signaling to the bone marrow via CCL8 (14) to replenish and maintain their populations, although an undefined mechanism for the transition of TRMs to TAMs has been observed (15). Through a combination of TLR and cytokine signaling, infiltrating MDSCs can also differentiate into TAMs and function as a source of TAM replenishment (46–49).

TAMs are identified and distinguished from MDSCs by the presence of characteristic surface markers that are shared with mature macrophages (22). Frequently described as M2-like macrophages, TAMs have distinct phenotypic and transcriptional characteristics that can be used to distinguish them from conventional M2-macrophages. Additionally, TAMs demonstrate marked immunosuppressive functionality not seen in the M2 macrophage population (50).

TRMs have an interesting role in tumorigenesis. Because they develop with the tissue, they are present long before any noticeable malignancy, but are thought to contribute to the early stages of tumor development (2). The contribution of various myeloid cell ontologies to tumor development and immunosuppression is highly debated (51), although myeloid cells recruited from the periphery seemingly have a more important role in propagating the growth and invasiveness of malignancies (17, 52). However, this might be a tumor-specific phenomenon, as evidence from breast cancer patients and murine models shows proliferating resident macrophages in the tumor contributing to the bulk of the myeloid compartment (19). Interestingly, there is some evidence that both populations may also play distinct roles in supporting tumor growth, and their origins bias their transcriptional networks (53). Therefore, it is possible that the developing tumor modulates both the tissue resident and infiltrating myeloid cell populations concurrently.

## Suppressive Mechanisms

The mechanisms of immunosuppression employed by TAMs and MDSCs are targeted toward inhibiting the activity of the adaptive immune system, namely T-cells, and NK cells. Suppressive myeloid cells do so by either direct cell-cell interaction with target cells, or through secreted factors. The mechanisms to suppress anti-tumor immune responses *in vivo* and have been extensively reviewed elsewhere (45, 50, 54–56). Briefly, they utilize four distinct functions to suppress T-cell mediated immunity: (1) signaling via the stereotypical inhibitory receptors PD-1 and CTLA-4 mediate leukocyte apoptosis and anergy (57–61); (2) depriving the local environment of nutrients necessary for T-cell activation and function (62–67); (3) generation of nitric oxygen and reactive nitrogen species, by iNOS expression, that induce T-cell exhaustion (12, 23, 62, 68, 69); (4) production of reactive oxygen species (12, 70). These mechanisms ultimately lead to a decrease in the effect and numbers of anti-tumor T-cells while enhancing the populations of tumor supporting regulatory T-cells (23, 24, 71, 72).

### Suppressive Programming

Stereotypically, STAT and PPAR signaling pathways are independently responsible for programming that drives suppressive functionality of myeloid cells (73, 74), but there are studies that describe their joint interaction in programming as well (75). STAT3 signaling in myeloid cells can be initiated by tumor derived factors, including IL-10 and lactate. Activation of STAT3 typically results in activation of SOCS to block intracellular inflammation cascades and initiate an “M2-like” state, complete with functional and phenotypical markers, such as ARG1 and CD206. More importantly, STAT3 activation also results in the production of factors that benefit tumor viability and invasiveness, such as VEGF, matrix metalloproteases, and IDO (76–78). With respect to MDSCs, STAT3 has been identified as a crucial factor for both their development and function. STAT3 is capable of modulating gene expression of anti-apoptotic proteins Bcl-xL, c-Myc, Cyclin D1, and others to promote cell survival. STAT3 also engages programs that prevent monocytic lineages from terminal differentiation to maintain an immature phenotype, a hallmark of M-MDSCs (27). Supporting the central role STAT3 plays in MDSC function, inhibition or deletion of STAT3 abrogates the function and development of MDSCs *in vivo* (79, 80).

STAT6 signaling also promotes a suppressive program in myeloid cells. IL-4 and IL-13 induce a cascade of phosphorylation events that eventually lead to phosphorylation and homodimerization of STAT6, translocation to the nucleus, and binding to the promoters for various “M2-like” genes, such as ARG1 and CD206. As is the case for STAT3, STAT6 can also bind to IFNγ-induced activation sites and repress the transcription of associated genes (81). One of the transcriptional targets of STAT6 is PPARγ, which augments the effect of the suppressive programming set in place by STAT6 (75, 82). Moreover, PPARγ also increases oxidative pathways that result in increased ROS production (83), among other suppressive pathways (84).

GCN2, an intracellular nutrient sensor, also regulates macrophage function and promotes the pro-tumorigenic phenotype of both TAMs and MDSCs by enhancing translation of the CREB-2/ATF4 transcriptional factor responsible for promoting their differentiation (64). Fundamentally the changes induced by these altered differentiation pathways results in a pro-tumorigenic response rather than mediating tumor elimination.

## Tumor-Associated Myeloid Cell Support of Tumor Growth & Progression

In addition to their role in aiding tumor immune evasion, TAMs and MDSCs also help orchestrate tumor progression. MDSCs remodel the extracellular matrix and promote blood flow to increase nutrient delivery via the production of various metalloproteases, cathepsins, and pro-angiogenic factors (24, 69). M-CSF can promote recruitment of peripheral myeloid cells to the tumor site and differentiate them into directors of angiogenesis (85). This distinct proangiogenic TAM subset, identified by surface TIE2 expression, secretes classic proangiogenic factors, such as VEGF proteins and SEMA4D (86, 87). These factors simultaneously retain anti-inflammatory functionality via autocrine and paracrine signaling through TIE2 (88). The combination of neovascularization and immune suppression can promote early dissemination of malignant cells (89), potentially through the breakdown of cadherin junctions between vascular endothelial cells (90). In some cases, the mobilization of TIE2^+^ macrophages is initialized as a response to chemotherapy, highlighting the complex systemic reaction to therapy.

Myeloid cell support of tumoral fitness isn't limited to the primary site of malignancy, as subsets of patrolling monocytes have been found to increase angiogenesis to distal metastatic sites (19). MDSCs can serve a similar role and “fertilize the soil” in pre-metastatic sites for malignant cells to settle. Through undefined mechanisms, MDSCs can be recruited to a premetastatic niche before TAMs and establish a nutrient-rich, vascularized, and immunosuppressive environment for tumors to seed (91, 92). Along the same lines, a subset of CCR2^+^ myeloid cells has also been associated with primary tumor recurrence (19), or re-fertilizing the soil for any remaining local or circulating tumor cells to grow.

## TAM/MDSC Identification Across Tumor Types

Identification of cells implicated in facilitating cancer growth is imperative for several reasons. Despite established knowledge that TAM/MDSC infiltration is associated with worse prognosis (93), it is clear that not all myeloid cells in the tumor microenvironment directly benefit the growing malignancy. Finding a defined population specifically associated with tumor aggressiveness or invasiveness can serve as a prognostic marker. Furthermore, chemotherapy is not a “silver-bullet” to diminish or deplete malignant cells. It results in changes to the local and distant environment that are not easy to predict without studying the effects *in vivo* or *ex vivo* (40). Beneficial off-target effects are possible, such as concurrently depleting myeloid cells from the tumor microenvironment (94). Some therapies, however, can exacerbate the suppressive actions of TAMs, MDSCs, and other local myeloid cells, reducing their *in vivo* efficacy (95–97). It is also unclear as to which myeloid cell subsets are most affected by the therapy. Defining the myeloid cell subsets that are resistant, or even retaliatory, to a particular therapy is crucial for response prediction. Lastly, defining the myeloid suppressive phenotype that is most associated with malignancy and most associated with therapy resistance brings therapeutic efforts one step closer to targeting a specific cell cluster that contributes to several requirements of the hallmarks of cancer (1, 98, 99).

Historically, identification of stromal contribution was achieved with immunohistochemistry and staining for a limited set of markers on serial sections. This practice, however, can be quite wasteful of precious biological specimens and data due to the limited number of concurrent stains that can be performed. As the definitions of all of the players in the tumor microenvironment are expanding exponentially, an expanded panel of markers must be employed to adequately study the TIME. Tissue analysis at single-cell resolution is allowing for discoveries of distinct myeloid cell phenotypes and connecting their gene and protein expression patterns to immunosuppressive and tumor-promoting mechanisms (98, 100). The myeloid compartment has vast heterogeneity in itself, even within monocyte/macrophage subsets (43). Commonly identified subsets are TAMs, monocytes, TRMs, and MDSCs. TAMs and MDSCs are the most interesting populations, as they seem to have the highest correlation to tumor progression and are typically present in the greatest quantities, compared to other immune cells (69). Within these populations are even more complex subsets. Technologies such as scRNAseq (101) and mass cytometry (102, 103) have created new definitions for these populations that highlight heterogeneity previously unappreciated by conventional flow cytometry, allowing for discoveries of rare cell populations. These technologies have also effectively outdated the standard classification scheme of M1- vs. M2-like phenotypes for macrophages. Standard M1/M2-like phenotypic markers should not be applied with absolute exclusivity, as many of the stereotypic genes that represent classical or alternative activation states can be co-expressed and even correlated with each other (43). Therefore, it is crucial to perform deeper statistical analyses to identify these smaller subsets that are more closely associated with the initiation, progression, and maintenance of the malignant niche, in addition to patient outcomes.

**Figure 1:**
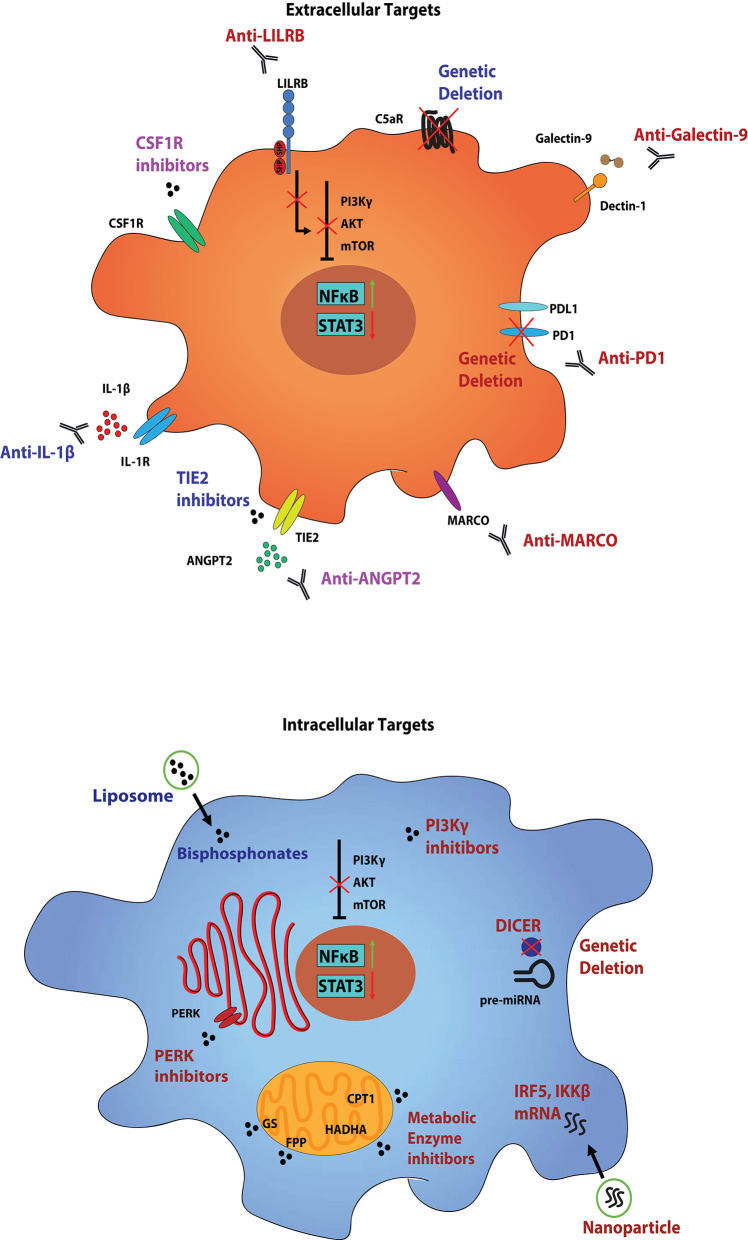
Myeloid cell populations within the TIME and strategies to target them. Tumor-infiltrating myeloid cells, such as MDSCs and TAMs, perform a variety of functions in order to keep the TIME a hospitable niche for tumor growth and progression. Mechanisms include direct secretion of growth factors to support the viability of the tumor, in addition to maintaining an immunosuppressive environment to prevent recognition by cytotoxic immune cells. These functional states are controlled by the growing tumor itself through various combinations of ligand-receptor interactions, and can be propagated by the tumor-associated myeloid cells. Several markers, both surface and intracellular, can be used to not only identify the individual populations of tumor-associated myeloid cells, but also as therapeutic targets. Therapies aimed at these targets generally serve to either deplete the individual clusters of cells from the TIME, or to reprogram them from pro- to anti-tumor states. Presented are conserved targets on MDSCs and TAMs across tumor types, although they exist in different combinations amongst various tumor-associated clusters. The simplified cell diagram on the top presents various surface targets to reprogram (red), deplete (blue), or a combination of both (purple), tumor-associated myeloid populations, and the simplified diagram on the bottom presents intracellular targets. While only a single cell diagram is portrayed, these strategies represent individualized therapies in targeting specific tumor-associated myeloid cell populations. While some receptors may overlap between populations, we hypothesize that a multifactorial approach is imperative to abolish myeloid cell support of tumor growth.

In defining the PD-1/PD-L1 (104) interaction and CTLA-4/CD80/86 (105), the search for novel immune checkpoints broadened into identifying new mechanisms that keep the adaptive immune cell out of the tumor environment and immunologically ignorant (2). More recently, myeloid cells in and around the tumor microenvironment have been recognized, as their utility for prognostication becomes more delineated. Generally speaking, TAMs, and MDSCs perform the same task of nurturing tumor growth among all cancers (106). The subset of culprit cells and the mechanisms by which they cloak or support the cancer can range. The surface markers of TAMs and MDSCs are not easily defined. Some markers of alternative activation are shared among TAMs and MDSCs, such as CD163^+^, CD68^+^ (40), or CD206^+^ (107), the same cells can also express markers of classical activation, such as CD169 and CD163 (107). Additionally, TAMs and MDSCs of different malignancies have different phenotypes, indicating differences in mechanisms of suppression, albeit with minimal conservation. Below, we highlight breast, lung, and central nervous system malignancies to address the myeloid cell heterogeneity, as these are the tumor models that have sufficient studies defining single-cell immune populations. For quick reference, immunosuppressive mechanisms discussed throughout the text are summarized in Figure 1 and Table 1. We have also summarized outstanding myeloid cell populations discussed in the text in Table 2.

**Table 1:** Immunosuppressive mechanisms employed by MDSCs and TAMs, as well as stereotypic programming that regulate the mechanisms.

	**Effect**	**Tumor type**	**References**
**Immunosuppressive mechanism**			
PD-1/PD-L1	T-cell exhaustion/suppression Myeloid cell suppressive programming	Glioma, Breast, Lung (non-small cell)	(45, 55, 58–61) (108, 109)
CTLA-4/CD80/86	T-cell exhaustion	Breast, Lung	(45, 58, 110, 111)
B7-H3 Receptor/B7-H3	T-cell exhaustion/suppression	Breast, Lung	(206, 207)
ARG	Environmental nutrient depletion	Breast, Lung	(45, 62, 63)
IDO	Environmental nutrient depletion	Breast, Lung	(55)
NOS	T-cell suppression	Breast, Brain, Lung	(12, 23, 45, 55, 62, 68, 69)
ROS	T-cell suppression	Breast, Brain, Lung	(12, 55)
**Immunosuppressive program**			
STAT	Inhibition of intracellular inflammation cascade in suppressive myeloid cells Anti-apoptosis in suppressive myeloid cells	Breast, Brain (GBM), Lung	(75–78, 81, 82) (27, 79, 80)
PPARγ	Inhibition of intracellular inflammation cascade in suppressive myeloid cells Metabolic reprogramming in suppressive myeloid cells	Lung, Breast	(74, 75) (83)

**Table 2:** Specific clusters of myeloid cells highlighted in the text are summarized here.

**Outstanding clusters**	**Hypothesized role**	**Cell type**	**Tumor studied**	**Species**	**Reference**
STAT3, B7H3, CSF1R, CCL3, SIGLEC1	Additional myeloid recruitment	TAM	Breast cancer	Human	(43)
PPARG, NRP2	T cell suppression	TAM	Breast cancer, lung cancer	Human, murine	(43, 106, 112)
PD-L1	T cell suppression	TAM	Breast cancer	Human	(107)
CD38	Tumor proliferation and migration	M-MDSC	Breast cancer	Human	(107)
TREM2, APOE, MARCO	Mature TAM markers; global immunosuppression; anti-apoptosis	TAM	Breast cancer, lung cancer	Human	(43, 112, 113)
IFITM1, SOCS3, TSPO	Global immunosuppression; ROS production and T cell suppression	MDSC	Breast cancer	Murine	(20)
LILRB2	Global immunosuppression	Monocyte-early TAM/MDSC	Lung cancer, GBM	Human	(106, 114)
CCL2, CCL3, CCL8, IDO1, IL1RN, IL4I1, NFKBIA, VISTA, LILRB4	Additional myeloid recruitment; global immunosuppression	Monocyte-early TAM	Lung cancer	Human	(113)
CXCL9, CXCL10, CXCL11, STAT3, CCR2, LILRB2, PDL1, IL4I1, IDO1	Global immunosuppression; T cell recruitment & suppression; chemotherapy resistance	TAM	Lung cancer	Human	(115)
IL10, TGFB2	Global immunosuppression; tumor progression	MDSC & infiltrating macrophage	GBM	Human, rat	(116–118)
TSPO	ROS production and T cell suppression	Infiltrating macrophage	GBM	Human	(116)
VEGFA, HIF1A	Tumor progression	Infiltrating macrophage	GBM	Human	(114)
PDL1, B7H3, CD86	T cell suppression	Microglia	GBM	Human	(114)
CCL3, TGFB2	Additional myeloid recruitment	MDSC	GBM	Human	(116)

*Several clusters overlapped between various malignancies*.

### Breast Malignancy

Without stratifying by breast cancer subtypes or stages, the myeloid landscape presented by different studies shows similarities. Notably, individual TAMs co-express both M1-like and M2-like associated genes along the same positive correlation trajectory (43, 107). Azizi et al. (43) identified TAM populations from human samples that expressed both classical and alternative activation markers, such as *CCL3* and *MARCO*, respectively, in addition to enrichment of signaling networks that are associated with each of the activation states. Highlighting a potential role for further recruitment of additional TAMs to the malignant site, one TAM cluster in the study by Azizi et al. (43) had distinctly enhanced expression of *STAT3, B7H3, CSF1R*, and *CCL3*. This same cluster also had upregulated *SIGLEC1*, which can serve as an independent predictor of poor prognosis [(14, 43), Supp.]. A separate TAM cluster in the same study was enriched in *PPARG* and *NRP2*, indicating distinct functional properties as a potential suppressor of T-cell activity through NRP2 (43, 119). Azizi et al. (43) further validated the individuality of the clusters and rejected the null hypothesis of unimodality across components that explain their variation.

Using scRNAseq information, Wagner et al. (107) detailed TAMs and MDSCs present in human breast cancer. A unique population of PD-L1^+^ TAMs and a population of MDSCs with high expression of CD38 is also identifiable among breast cancer samples (107). Notably, CD38 has been found to aid the proliferation and migration of tumor cells and is also independently associated with the establishment of an immunosuppressive environment, even when expressed on M-MDSCs isolated from peripheral blood (120–122). As a note of caution, studying peripheral blood immune cells as biomarkers for diseases comes with its own challenges, as PBMC phenotypes don't necessarily agree with tumor-infiltrated immune cells (106). The complexity and heterogeneity of intra-tumoral myeloid cell populations is not well-represented by peripheral myeloid cells, possibly due to the effect of local tumor-associated signaling, therefore care must be taken when associating peripheral cells to the local disease. However, locally expressed CD38 can bypass disinhibition from PD-1/PD-L1 targeted therapy (123).

The TAM population in breast cancer studies seems to be the most mature cell population, defined by a signature defined by several factors, such as *TREM2, APOE*, and *MARCO* (43). All can be used as phenotypic markers of mature myeloid populations, such as macrophages, but TREM2 can serve as a functional marker of an anti-apoptotic state (124). Similar populations of TAMs are described in other cancers later (14, 112). Several studies showed the presence of undifferentiated monocyte populations within the breast TIME. Azizi et al. (43) described several monocyte populations with no enrichment of immune gene sets in addition to several other populations that are on track to dendritic cell differentiation. Likewise, Wagner et al. (107) described a border of monocytes to wall off the TAMs within the tumor core.

In murine models of breast cancer by Alshetaiwi et al. (20) MDSCs can be distinguished with scRNAseq. However, their identification presents a sizable challenge, as they do generally do not form distinct clusters by standard informatics analyses. With deeper analysis, they are distinguishable from other myeloid cell populations by their own transcriptional signature (20). Most notable in their transcriptional signature is the dramatic upregulation of *IFITM1* and *SOCS3*, marking their suppressive programming, in addition to *TSPO* (translocator protein) when compared to other myeloid cell clusters, highlighting their functional role in the TIME. TSPO is a mitochondrial membrane protein that, when activated, results in a respiratory burst and generates reactive oxygen species from myeloid cells, subsequently causing inhibition of T-cell activity (125). Unfortunately, no studies to date have evaluated the phenotypes of individual MDSC clusters to differentiate their functional roles in the TIME, although it is hypothesized that distinct clusters do exist (126).

Taken together, phenotypically distinct populations of TAMs/MDSCs have different functional responsibilities within the TIME in breast cancer. Notably, the majority of these suppressive cells are more mature TAMs, rather than MDSCs. Yet to be determined is the ontogeny of TAMs, i.e., whether they are the product of MDSC maturation or monocyte differentiation.

### Lung Malignancy

Normal lung tissue is rich in immune cells responsible for eliminating foreign bodies and infections, therefore it is important to segregate TAM/MDSC populations from the normal lung myeloid populations for correct analysis. In adenocarcinoma, TAMs may have expression networks that make them more readily identifiable from normal myeloid cells, but deeper analyses like scRNAseq is required in order to differentiate their signatures and identify distinct populations (112, 113). TAMs in a later stage of macrophage differentiation within lung adenocarcinoma are distinguishable from resident myeloid cells via concurrent expression of *TREM2, MARCO*, and *APOE*, as mentioned earlier. As in other tumors, the TAMs from early lung adenocarcinoma express M1- and M2-like markers, including *HLA-DR* and *CD163*, respectively. Importantly, subsets of TAM populations in non-squamous cell lung cancer (NSCLC) show an enrichment of *PPARG* expression that can initiate anti-inflammatory transcriptional networks that propagate immune ignorance (127, 128), differentiating them from both normal lung macrophages and peripheral myeloid cells (106, 112, 115, 129). Zilionis et al. (106) also describe a population of tumor-infiltrating monocytes that express anti-inflammatory-like markers, such as *LILRB2*, a potent activator of the STAT6 signaling network. As this population has comparably low *CD14* expression, we speculate that this population of monocytes could represent newly-trafficked cells [(106), Supp.] that display immunosuppressive functionality early in the TAM differentiation process. This supports the notion that tumoral recruitment of suppressive cells happens early and at a systemic level. Our group has shown that the murine homolog to LILRB2, PIRB, can regulate the entire network of suppressive functionality of myeloid cells, making the expression of LILRB2 an interesting therapeutic target (130). Additionally, we have shown that targeted therapy against LILRB2 on tumor-infiltrating myeloid cells can reverse their suppressive fate initiated by the malignancy and diminish lung cancer tumor burden in murine models (131).

Tumor associated myeloid cells in lung cancer have the ability to further recruit new myeloid cells, as seen in other cancer types. Lambrechts et al. (113) describe the heterogeneity of immune cells within NSCLC, and describe a particular myeloid cell compartment that is enriched in several genes that recruit more immune cells to its location, such as *CCL2, CCL3*, and *CCL8*, in addition to *IDO1, IL1RN* (132). The same cluster exhibits high expression of *IL4I1, NFKBIA, VISTA*, and *LILRB4*. Like LILRB2, LILRB4-mediated ITIM signaling has a strong effect on the anti-inflammatory phenotype of myeloid cells (133), and we hypothesize that LILRB4 could act as a central regulator of the immunosuppressive cascade network in this myeloid cluster, as Deng et al. (134) showed a significant decrease of NFKBIA (IκB) at the protein level, following genetic ablation of LILRB4 in myeloid cells (135). The additional correlation to VISTA within the same cluster is of particular importance, as VISTA is proving to be an attractive target to prevent inhibition of T-cell cytotoxicity (136). In concert, this network would presumably directly program newly recruited myeloid cells to a suppressive state and add to the immunosuppressive border surrounding the growing malignancy.

Clusters of suppressive myeloid cells can incorporate other cell types to augment their effect. A population of macrophages has been shown to induce T-regulatory cells to further fortify the immune barrier to cancer recognition (115). This macrophage cluster expresses markers of T-cell recruitment, such as *CXCL9, CXCL10*, and *CXCL11*, but the cluster is also enriched for anti-inflammatory-like genes, such as *STAT3, CCR2*, and *LILRB2* [(115), Supp.]. Most importantly, the same cluster is extraordinarily enriched for *PDL1, IL4I1*, and *IDO1*—genes heavily implicated in suppression of cytotoxic T-cell activity and induction of T-regulatory cell programming (137–142). According to Maynard et al. (115), this cell population is expanded in patients that show progression of malignancy on therapy, highlighting a crucial mechanism for therapy failure that corroborates previous work (143). This demonstrates another role of myeloid cells in tumoral viability—creating a hospitable environment for recurrence. While the entire population of myeloid cells is frequently targeted for cancer therapeutics (144), it's clear that more efficient strategies are needed. From the study by Lambrechts et al. [(113), Supp.], there does not appear to be any one particular myeloid cluster that has outstanding expression of PD-L1, PD-1, or B7-H3, underscoring the relevance of the other strategies employed by TAMs to keep the adaptive immunity at bay.

In summary, the lung cancer studies show off the power of deep analysis of the tumor microenvironment. Even in the case of the TAM compartment, which is frequently depicted as a single cell type, there is substantial heterogeneity in cell types that seemingly assume different roles to protect and contribute to the tumor growth. This also underlines a key aspect of immunotherapy targeted against the tumor microenvironment: it is unlikely a single therapeutic would have the capability to transform or reprogram all involved cells—in this case, TAMs/MDSCs. While targets such as PD-1/PD-L1 or CTLA4/CD80 are important, these mechanisms address just one mechanism of TAM-mediated suppression, and a downstream effector, which could explain the limited clinical benefit.

### Central Nervous System Malignancy

Central nervous system (CNS) malignancies account for a small percentage of all diagnosed cancers (145), but they are frequently associated with abysmal prognoses. The resident immune system of the CNS, namely the microglia, are established contributors to CNS malignancies (146), but there are several other phagocytic myeloid cell populations in the CNS that are also, if not more so, implicated in a poor prognosis of the most aggressive form of CNS malignancy, glioblastoma multiforme (GBM). Perivascular, meningeal, and choroid plexus macrophages of the CNS have generally been overlooked as contributors to GBM (147, 148), but the involvement of bone marrow-derived myeloid cells has recently been established, and even positively correlated, to poor outcomes in GBM models (116, 149). As seen in the previous cancer studies, GBM TAMs co-express M1- and M2-associated markers, again making simple surface phenotyping of cells rather difficult, and creating the need for mechanism and pathway analysis (116). Invading peripheral myeloid cells show a greater suppressive potential than do microglia, marked by increased expression of *IL10* and *TGFB2*—potent inducers of T-regulatory cells (12, 43)—compared to the resident immune cells (116, 117). Likewise, peripheral myeloid cells were also enriched in genes involved in the citric acid cycle and *TSPO* compared to the resident microglia, resembling TAMs that we speculate to directly inhibit T-cell functionality mentioned previously in the Breast Cancer section [(116), Supp.].

Unfortunately, current scRNA-seq studies of the TIME in GBM use consensus clustering only to distinguish the roles of microglia and peripheral macrophages. This method limits the resolution and only allows for the evaluation of two myeloid cell clusters. Despite this, Muller et al. (116) describe myeloid cell heterogeneity that is the result of their spatial relationship with the malignancy, suggesting that suppressive myeloid cells perform different roles according to their physical location. Likewise, Darmanis et al. (114) show that macrophages make up the majority of myeloid cells within the tumor core and microglia make up the myeloid population of the surrounding stroma. The macrophages in the core seemingly contribute more to the overall viability of the tumor via their expression of *VEGFA* and *HIF1A*, while the juxtatumoral microglia serve as the main masqueraders of the malignancy with increased expression of *PDL1, B7H3, CD80*, and *CD86* (114). The myeloid cells within the tumor core are also the main source of *LILRB2* expression, offering a selective target for reprogramming a significant cell population for maintaining tumoral viability. Also interesting is that the majority of *LILRB2*-expressing myeloid cells do not co-express *MARCO*, a pattern recognition receptor enriched on TAMs (150); we speculate that these cells could be MDSCs (114). Most GBM-associated myeloid cell populations are involved in recruiting additional immunosuppressive myeloid cells, marked by exorbitant expression of *CCL3* and *TGFB2* in numerous GBM specimens (116). Combined expression of *CCL3* and *TGFB2* in a variety of bulk tumor samples from tissues of different origin is strongly associated with the local presence of MDSCs, despite the difficulty in their identification (151). More importantly, high expression of the combination is associated with a worse overall median survival in high grade glioma, referenced in multiple data repositories (152).

While there are limited studies that recognize the presence of MDSCs, and specifically analyze heterogeneity of MDSCs, in models of CNS malignancy, it is imperative that we discuss them in this context. MDSCs have been detected in the tumor microenvironment and play a significant role in tumor progression (153). They do not exist in healthy CNS tissue outside of the context of malignancy (149, 153). Alban et al. (118) use MDSC infiltration in GBM as prognostic markers and indicate a hazard ratio of 4.7 (1.69–13.4) when comparing overall survival of patients with high MDSC GBM infiltration to low infiltration. Under the assumption that all M-MDSC populations that infiltrate GBMs are programmed into the same functional state, their role is to secrete IL-10 and TGF-β, just like their macrophage counterparts. The presence of these cytokines is correlated to overall stage of the malignancy [(118, 149), Supp., (154)], indicating that there is most likely a dose effect as a greater amount of MDSCs in the local environment is correlated to staging as well.

In addition to the local involvement of suppressive myeloid cells, the peripheral differential cell count offers insight to prognosis of GBM patients (118, 149). MDSCs in the periphery are heavily implicated in higher grade, more aggressive CNS malignancies. Peripheral MDSCs have a strong positive correlation with worse prognoses in GBM patients, and the converse is true as well. Alban et al. (118) showed that, after surgical resection of GBMs, patients with increasing fractions of MDSC populations had inferior survival time, compared to those of decreasing MDSC fractions. A cohort of newly diagnosed patients in the study received standard-of-care adjuvant therapy (155), but the expansion of M-MDSCs were variable, indicating a potential difference in activation of myeloid cells following chemo- or radiotherapy (156, 157). Additionally, a number of studies show increased peripheral MDSC counts in subsets of patients who received dexamethasone perioperatively, indicating a potential confounder, or contributor, in correlating overall survival with MDSC levels (118, 148, 149).

GBM is well-known to be an extraordinarily heterogeneous malignancy, making it very difficult to target with “off-the-shelf” therapy. However, it is striking to see that even across the heterogeneity of malignancies from different patients, the myeloid cell clustering, and signaling networks seem to remain conserved (114). Manipulating the programming of both bone marrow-derived myeloid cells and resident microglia is important in regulating the entire network of immune suppression and pro-tumor functionality. While microglia appear to be attractive targets for the popular therapies targeting PD-L1 or B7 family of proteins, involvement of the peripheral immune system within the tumor microenvironment is more closely associated to prognoses and should also be considered for immunomodulation. Whether the infiltrating TAMs, the malignancy itself, or a combination of both is causing the suppressive programing of the microglia remains to be determined. Table 1 details the pathways and receptors that mediate immunosuppression along with the specific effect and tumors impacted by the signaling pathway.

## Methods to Prevent Myeloid Cell Contribution to Cancer Growth

Currently, there are two main strategies for manipulating tumor associated myeloid cells: depletion and reprogramming. Depletion involves broad, systemic targeting of myeloid cells, although newer, more specific approaches are aimed at depleting only the myeloid cells that are specifically involved with the malignancy (40). The therapeutic strategies are summarized in Table 3, along with recent clinical trial information.

**Table 3:** Therapies used to reprogram tumor associated macrophages and MDSCs.

**Target**	**Therapy/Treatment**	**Clinical Trials**	**References**
BTK/ PI3Kγ	Small molecule BTK inhibitor: Ibrutinib Small molecule PI3K inhibitor: IPI-549	NCT03379428 NCT02403271 NCT02321540 NCT02950038 NCT02403271 NCT03535350 NCT03961698 NCT03719326	(158) (159)
LILRB	Anti-LILRB2 antibody	N/A	(131)
C5a/C5aR	C5aR genetic deletion	N/A	(160)
Dectin-1 (CLEC7A)/Gal-9	Anti-Gal-9 antibody	N/A	(161)
CSF-1/CSF-1R	Small molecule CSF-1R inhibitor: BLZ945	NCT02829723	(162)
IL-1β	Anti-IL-1β antibody	NCT02900664 NCT03742349 NCT03447769 NCT03968419 NCT03631199 NCT03626545 NCT03064854	(163)
HIF1α/β	HIF1 genetic deletion	N/A NCT01036113	(164)
ANGPT2/TIE2	Small molecule TIE2 inhibitor: Rebastinib Anti-ANGPT2 antibody: Nesvacumab	NCT02824575 NCT03717415 NCT03601897 NCT01688960	(164) (165)
PERK (UPR)	Inhibitor of unfolded protein response: Tauroursodeoxycholic acid (TUDCA)	N/A	(166)
Glutamine Synthetase (GS)	Methionine Sulfoxamine	N/A	(167)
CPT1 (FAO enzyme)HADHA (FAO enzyme)	Small molecule CPT1 inhibitor: Etoximir/Perhexiline Small molecule HADHA inhibitor: Ranolazine	N/A	(168) (169) (170)
FPP	Small molecule FPP inhibitor: Zoledronic Acid	NCT02347163 NCT00295867 NCT00320710 NCT03664687	(171) (172) (173)
MARCO	Anti-MARCO antibody	N/A	(150)
IRF5IKKβ	Nanoparticle encapsulated mRNAs	N/A	(174)
DICER	DICER genetic deletion	NCT01353300 NCT00565903	(175)
PD-1/PD-L1	PD-1 genetic deletionAnti-PD-1 antibody	NCT04173325 NCT03414684 NCT03925246	(108) (109)

### Depletion

Strategies to deplete myeloid cells from the TME include mechanisms to prevent myeloid cell trafficking to the malignancy or initiate apoptosis. Tumoral recruitment and expansion of bone marrow-derived myeloid cells occurs through a CCR2-CCL2–dependent signal and, along with increasing serum levels of CCL2, is independently associated with worse prognosis. Disruption of CCR2 signaling prevents the recruitment and development of suppressive myeloid cells, while suppressing tumor metastasis and prolongs survival across several cancer models (16, 176, 177). Importantly, disrupting CCR2 signaling also reduces TAM/MDSC recruitment to premetastatic niches (16).

Antagonizing the CSF1–CSF1R axis is an interesting approach as it disrupts several mechanisms for therapeutic effect. Blocking the axis can disrupt localization of suppressive TAMs to the site of malignancy (178) as well as reprogram TAMs for anti-tumor activity (162), in addition to preventing the conversion of M-MDSCs to TAMs (12). JNJ-28312141, a CSF1R inhibitor, depleted F4/80^+^ TAMs in a subcutaneous H460 human lung tumor xenograft model and increased plasma CSF1, a potential biomarker in CSF1R inhibition (179). Biologics have also been studied in this regard—RG7155, a monoclonal CSF1R antibody, greatly reduced F4/80^+^ TAMs in animal models of colon cancer. RG7155 showed promise in human applications as well, as it induced apoptosis of CSF1R^+^CD163^+^ macrophages in patients with diffuse type giant cell tumor tissue (Dt-GCT) (178). However, as CSFR1 blockage with pexidartinib has proven to be ineffective in patients, targeting the CSF1–CSF1R signaling axis might have limited applications (180). Combination therapy of CSF1R blockade with immune checkpoint blockade is currently ongoing in a solid malignancy clinical trial (Trial # NCT02713529).

Targeting CD38 is proving to be a good strategy for antibody-mediated depletion in some cancer models. CD38^+^ MDSC populations are expanded in cancer patients and can even serve as an escape mechanism after PD-1/PD-L1 therapy. Daratumumab, a CD38 antagonist antibody, can deplete immunosuppressive myeloid cells from circulation, as well as serve as an independent therapy for CD38^+^ myelomas. CD38 antibody therapy initiates apoptosis via antibody-dependent cell-mediated cytotoxicity and complement-dependent cytotoxicity. Other suppressive cell types, such as T-regs, are also sensitive to anti-CD38 treatment (122, 123, 181).

Liposomal delivery of dichloromethylene biphosphonates is another effective method to deplete tumor associated myeloid cells, as it deposits its payload directly into the intracellular space. Liposomes are enclosed multifunctional structures that consist of one or more phospholipid bilayers surrounding a hydrophilic core. This organization allows for hydrophobic therapies to associate with the lipid bilayer, and hydrophilic therapies, including genetic material such as RNA, DNA or siRNA, to be carried in the core. Clodronate and other bisphosphonates are a class of drugs typically used for the treatment of osteolytic bone disease and osteoporosis by inhibiting bone resorption, as they specifically target the phagocytic cells involved (182). By encapsulating clodronate in liposomes, clodronate can be delivered to the tumor site where it is phagocytosed by macrophages, ultimately initiating apoptosis. However, these effects have only been shown *in vitro* and animal models (183–185).

An interesting, albeit controversial, aspect of MDSCs in the TIME is the effect of chemotherapies on MDSC quantities and suppressive programming. 5-fluorouracil (5-FU) and gemcitabine were able to induce apoptosis and deplete MDSCs in both spleens and tumors in 4T1 murine breast cancer model. Moreover, both 5-FU and gemcitabine can activate caspase-1 and induce IL-1β production via the NLRP3 inflammasome pathway (186, 187). Evidence points to conflicting effects of IL-1β with respect to the TIME. While some studies show a beneficial effect of increased IL-1β in the TIME (64), others show that blockade of IL-1β signaling can prevent immunosuppressive cell recruitment (163). Other secondary effects of chemotherapy on the immune system are discussed in depth elsewhere (40, 54).

### Reprogramming

The tumor microenvironment can polarize TAMs to an immunosuppressive M2-like functional state, leading to enhanced tumor growth, progression, and metastasis. Besides depleting TAMs and MDSCs, myeloid cells can be reprogrammed toward a pro-inflammatory state by direct intervention via small molecules and antibodies targeting key receptors. Two reprogramming strategies can be used—blocking a receptor that normally transduces an inhibitory intracellular signal, or using an exogenous ligand to activate a receptor that stimulates pro-inflammatory intracellular cascades (188). Despite its success in diminishing tumor burden, pro-inflammatory agonist therapy is frequently associated with systemic toxicity (189, 190), therefore, we will discuss the former strategy.

#### Surface Targets

In some cases where disruption of the CSF1-CSF1R signaling axis is unsuccessful in depleting TAMs, antagonism of CSF1R signaling can reprogram TAMs away from an M2-like state. Using glioma xenograft models, Pyonteck et al. (162) describe how CSF1R antagonism did not decrease TAM numbers nor did it alter their CSF1R expression pattern. However, inhibition of AKT phosphorylation and M2-related gene expression, such as *ARG1* and *CD206*, indicated that CSF1R antagonism initiated a functional shift to a pro-inflammatory state to block glioma progression (162).

In a murine pancreatic ductal adenocarcinoma (PDAC) model, crosstalk between B-cells and FcRγ^+^ TAMs resulted in an M2-like phenotype through Bruton's tyrosine kinase (BTK) activation in a PI3Kγ-dependent manner. Using the BTK inhibitor ibrutinib, PI3Kγ inhibition in PDAC tumor-bearing mice reprogrammed TAMs toward an M1-like state and increased CD8^+^ T-cell cytotoxicity to slow PDAC tumor growth (158). PI3K is a critical switch to promote suppressive activity in macrophages, as PI3K signaling via AKT and mTOR inhibits NFκB to promote M2-like functionality in TAMs. Conversely, inhibiting PI3K prevents C/EBPβ activation and disinhibits NFκB to induce a pro-inflammatory phenotype. Combining PI3K blockade with anti-PD-1 therapy can promote tumoral T-cell infiltration to slow tumor growth and enhance survival in tumor-bearing mice (159).

Signaling pathways that activate NFκB to initiate pro-inflammatory functionality represent valuable therapeutic strategies. Our group found that PIRB/LILRB signaling pathways can function as crucial regulators of NFκB activity. Ablation of PIRB in MDSCs forced a transition to an M1-like phenotype, resulting in decreased suppressive function, T-reg activation, tumor growth, and metastasis (130). PIRB^−/−^ monocytes expressed stereotypic markers of inflammatory functionality, such as increased iNOS, TNFα, with decreased IL-10 and ARG1 when compared to WT monocytes. PIRB^−/−^ MDSCs also demonstrated increased ERK, MAPK, and NFκB activation upon LPS stimulation, and enhanced IFNγ-related inflammatory responses. LILRB2—the human ortholog to murine PIRB—blockade via monoclonal antibodies favored the activation of NF-κB and STAT1 and the inhibition of STAT6 activation by IL-4. *In vitro*, we observed decreased levels of CD14, CD163, CD16, and DC-SIGN in A549-derived macrophages cultured in the presence of αLILRB2 antagonist antibodies. Humanized MISTRG (M-CSF^hi^, IL-3/GM-CSF^hi^, and TPO^hi^) mice treated with αLILRB2 antibodies to reprogram human macrophages to a M1-like classically activated phenotype. Our group has also generated BAC-transgenic mice expressing LILRB2 for various studies. Recently we showed that αLILRB2 antibody therapy had a synergistic effect when combined with αPD-1 therapy to diminish tumor burden in a lung cancer model with BAC-transgenic LILRB2 mice, while simultaneously suppressing MDSC and T-reg infiltration into the tumor site (131).

High Dectin-1 and the novel Dectin-1 agonist Galectin-9 expression were found in the TME of PDAC bearing mice. Dectin-1 is a c-type Lectin expressed mainly on macrophages and other myeloid-monocytic lineage cells. It is postulated that Dectin-1 ligation in TAMs leads to immunosuppression, thereby promoting PDAC growth. Dectin-1 does not have direct pro-tumorigenic effect on transformed PDAC but its deletion in tumor infiltrating macrophages induced immunogenic reprogramming. Similar to the outcome of Dectin-1 deletion, Galectin 9 neutralization enhanced intra-tumoral T-cell activation in PDAC (161).

MARCO (macrophage receptor with collagenous structure) is a scavenger receptor found on M2 immunosuppressive TAMs. We discussed the presence of this receptor in TAMs across multiple tumor types. Conditioned medium from cultured B16 melanoma cells and IL-10 stimulated culture resulted MARCO expression on M0 bone marrow derived macrophages (BMDM). Treatment with anti-MARCO antibodies decreased tumor sizes, increased M1-like, and decreased M2-like TAM populations in the TIME in 4T1 breast cancer and B16 melanoma mouse models. The TIME displayed decreased immature macrophages, increased CD4/T-reg cell ratio, and an upregulation of M1-like genes such as *TNF, IL-1*β, *NOS2*, and a downregulation of *IL-10* suggesting polarization of TAMs to a more inflammatory phenotype (150).

Last but not least, the PD-1/PD-L1 axis is one the best studied and most clinically successful checkpoint inhibitors. In cancer, the PD-1/PD-L1 axis is best known for T cell regulation. Previously, macrophages were known to express PD-1 during pathogenic infections (191, 192). Since then, it was discovered that TAMs can also express high levels of PD-1, with increasing levels over time in murine models and higher expression in increasing human cancer disease stage. PD-1/PD-L1 blockade *in vivo* increased PD-1^+^ macrophage phagocytosis activity and reduced tumor growth in murine colon carcinoma models (108). A more recent study showed that PD-1 ablation or blockade with monoclonal antibodies prevented the accumulation of granulocyte/macrophage progenitors under cancer driven emergency myelopoiesis. Interestingly, PD-1 deficient myeloid progenitors also had increased cholesterol synthesis which is required for the differentiation of inflammatory macrophages. Additionally, PD-1 ablation on myeloid cells decreased tumor growth more effectively than T-cell specific PD-1 ablation in a murine fibrosarcoma and melanoma models (109). Cumulatively, PD-1/PD-L1 blockade or ablation on myeloid cells promotes phagocytosis in macrophages, reprogramming of myeloid progenitors and even furthers myeloid differentiation via metabolic pathways. None of the aforementioned single-cell studies show exceptional levels of PD-1 on myeloid cells, but that does not exclude it from being a potential target for diminishing immunosuppressive phenotypes of myeloid cells.

#### Soluble Targets

C5a is a protein fragment released from cleavage of complement C5 that may be involved with PMN-MDSC recruitment. In one study, C5a was found to enhance tumor growth and inhibit CD8 T-cell mediated cytotoxicity by recruiting PMN-MDSC (CD11b^+^Gr1^+^) to the tumor microenvironment. C5a also enhanced PMN-MDSC's suppressive capacity by increasing the production of reactive oxygen (ROS) and nitrogen species (RNS) which inhibits CD8+ T cell response (193). Ablation of C5aR reduced the ratio of PMN-MDSC to M-MDSC in tumor bearing mice compared to wild type mice. C5aR blockade is a potential strategy to modulate the tumor microenvironment by preventing the recruitment of immunosuppressive PMN-MDSC (160).

IL-1β, a proinflammatory cytokine, is a potential target for macrophage reprogramming because it impacts CSF1/CSF1R signaling. In early tumor progression models using 4T1 cells in Balb/c mice, IL-1β acts as a master cytokine, exhibiting both pro- and anti-tumoral functionality (163). IL-1β recruited CCR2^+^ inflammatory monocytes to the tumor site through the induction of CCL2 but also promoted the differentiation of these monocytes into immunosuppressive macrophages by inducing CSF1. IL-1β deficient mice displayed significant reduction in inflammatory monocytes recruitment and macrophage differentiation. Combination therapy of αIL-1β and αPD-1 completely abrogated breast tumor progression (163).

#### Microenvironment

Besides cell surface receptors, cytokines and chemokines, the oxygen level in the tumor also affects the microenvironment. Tissue hypoxia develops as tumor cells proliferate until oxygen demand overwhelms the supply. To restore oxygen to the microenvironment, malignant cells initiate a hypoxic response to drive a more aggressive phenotype, promoting angiogenesis, cell proliferation, self-renewal, and other pro-tumoral programs. Two master regulators of hypoxia in cells are HIF1α and HIF2α. TAMs within this hypoxic environment are more strongly associated with M2-like functionality (194), and HIF2α ablation in TAMs resulted in a more favorable outcome in models of hepatocellular carcinoma and colitis associated colon carcinoma (164).

Just as oxygen levels affect the TIME, tumor vascularization also plays a role. The angiopoietin (ANGPT2)/TIE2 kinase signaling axis is essential to angiogenesis. TIE2 can be found on a subset of pro-angiogenic macrophages (TIE2^+^ macrophages) and promote tumor angiogenesis and tumor metastasis. Rebastinib, a TIE2 kinase inhibitor, suppressed the infiltration of TIE2^+^ macrophages to the tumor site in the PyMT mouse model of breast cancer (195). Another study showed that vascular endothelial production of ANGPT2 recruited TIE2^+^ macrophages to the tumor and the inhibition of ANGPT2 binding suppressed TAM recruitment (165).

Recent studies have attempted to explain why tumor-associated immunosuppressive myeloid cells cannot simply be binned in an M1/M2-like dichotomy, and Mohamed et al. (166) describe ER stress as a potential mechanism. Undefined tumoral signaling causes an upregulation in the unfolded protein response of MDSCs, leading to an activation of the intermediate media PERK and NRF2 drive the immunoregulatory phenotype. PERK ablation led to a reprogramming of MDSC functionality, specifically, to initiate a type I interferon anti-tumor response. More importantly, similar anti-tumor effects can be achieved with the exogenous administration of PERK inhibitors (166).

#### Metabolism

Recent studies have shown that immunometabolism plays a very important role in the regulation of macrophage function in the tumor microenvironment. The metabolic profile of TAMs determines their status as pro- or anti-tumoral effector cells. M1-like macrophage metabolism is generally characterized with increased glycolysis, fatty acid synthesis, and a truncated TCA cycle whereas M2-like macrophage metabolism is skewed toward fatty acid oxidation (FAO) and the TCA cycle (196–200). For example, tumor-derived lactate induces an M2-like state in macrophages, measured by the induction of the M2-related genes *VEGF, RELMA, MGL1*, and *MGL2*. Lactate can also promote the expression of ARG1 and stabilize HIF1α–key functional elements of a suppressive macrophage (201). Preventing the metabolic profile initiated by lactate using a small molecule inhibitor may reduce the presence of immunosuppressive myeloid cells (77). Similarly, methionine sulfoxamine, a potent inhibitor of glutamine synthetase, skewed M2-polarized macrophages toward an M1-like state characterized by reduced intracellular glutamine and increased succinate to promote glycolysis (167).

However, promoting glycolysis in macrophages of the TIME is a risky endeavor as cancer cells also preferentially use glycolysis as an energy source, according to the Warburg effect. Therefore, targeting a metabolic pathway that inhibits tumor progression while simultaneously promoting an anti-tumor immune response would be an attractive strategy. FAO is one potential pathway, as it is the defining metabolic program of M2-like macrophages. FAO inhibition can impair the proliferation of leukemia cells (168) and reduced cellular ATP and viability in glioma (169). In multiple tumor models, tumor infiltrating MDSC were found to have increased fatty acid uptake and activated FAO (170). Etomoxir, a pharmacologic inhibitor of FAO, decreased the overall metabolic activity of MDSCs, their ability to prevent T-cell proliferation, and production of critical cytokines that maintain the induction and differentiation of MDSCs. Tumor-bearing mice treated with etomoxir and a related inhibitor, ranolazine, showed delayed tumor growth attributable to increased T-cell mediated cytotoxicity (170).

As previously mentioned, liposomal delivery of bisphosphonates can be used to deplete macrophages via apoptosis. Zoledronic acid (ZA) is a bisphosphonate containing a double nitrogen group. It inhibits the active site of the enzyme farnesyl pyrophosphate synthase in the mevalonate pathway, which is critical for isoprenoid and cholesterol synthesis (171). ZA also has a direct proapoptotic effect on tumor cells and reduces their metastatic potential (172). TAMs were significantly reduced in a TUBO cell murine mammary tumor model. Peritoneal macrophages and TAMs in ZA-treated mice displayed enhanced M1-like markers, shown by nuclear translocation of NFκB, NOS expression, and NO production (173).

#### Genetic Modification

Gene therapy is a unique strategy to polarize TAMs. Zhang et al. (174) describe using *in vitro*-transcribed mRNA encoding IRF5 and its activating kinase IKKβ encapsulated in nanoparticles to reprogram TAMs in models of ovarian cancer, melanoma, and GBM. The nanoparticles were engineered with D-mannose on the surface to efficiently and specifically target the mannose receptor CD206^+^ on TAMs. Upon mRNA uptake, TAMs adopted a tumor-clearing, pro-inflammatory profile (174).

Similarly, endogenous RNA processing mechanisms can be exploited to reprogram TAMs. MicroRNAs (miRNA) are a class of small non-coding RNAs that negatively regulate RNA transcription and transcript levels through a sequence dependent mechanism. Normally, DICER, an RNAse-III enzyme, processes hairpin-shaped precursor miRNAs into mature miRNAs (202). Baer et al. (175) describe conditional deletion of DICER in TAMs to prevent maturation of miRNAs that otherwise inhibit M1-like functionality, rewiring the cells toward a pro-inflammatory state characterized by the activation of IFNγ and STAT1 signaling. Moreover, DICER-deficient TAMs promoted the recruitment of cytotoxic T cells that completely eradicated tumors in mouse models when combined with PD-1 checkpoint blockade (175). A summary of these methods, specific targets, and ongoing clinical trials to target them is provided in Table 3.

## Conclusion, Questions, Limitations

Emerging techniques such as scRNAseq and mass cytometry have allowed for enhanced analyses of previously uncharacterized cell subsets in the tumor immune microenvironment, offering new avenues for discovering potential novel therapeutic targets and pathways that support tumor progression. Although these have not translated into the clinic yet, there is optimism that greater understanding of the tumor immune microenvironment and associated immunomodulatory mechanisms will allow for targeted therapeutic strategies to improve patient survival. While the tumor-associated myeloid cell population collectively functions to support the growing malignancy, subsets of the population are driven by assorted environmental cues that induce different functional programs. Different subsets of tumor-associated myeloid cells can directly contribute to the viability of the tumor, prohibit recognition of the tumor by the adaptive immune system, and drive chemotherapy or immunotherapy resistance. The questions left to be answered are: what combinations of signals cause the heterogeneity within the microenvironment and do they originate from the parenchyma, stroma, or both? What effect does chemotherapy or immunomodulation have on the various populations? Is there a specific population that is correlated with local or distal recurrence? For any of these cases, is one subset enough to drive any of these phenomena, or is the collection of these subsets necessary? Is there a combination of therapies that would be most effective in eradicating these detrimental subsets?

Defining previously uncharacterized subsets of immune cells by single-cell analyses is crucial to the understanding of tumor biology, but *in situ* cell relationships also require attention. Loss of tissue architecture is a major limitation to suspension-cell-based assays, such as scRNAseq and suspension mass cytometry, thereby discounting important spatial information that comes from delineating cell-cell interactions. Several of the studies referenced above underscored heterogeneity of myeloid cell phenotypes based on their physical orientation to the tumor—within the tumor or surrounding the periphery of the tumor (18, 107, 116, 203). The location in which cells are found also dictates their functional role in the development of the malignancy, as juxtatumoral immune cells most likely serve as a suppressive barrier to cloak the malignancy, while intratumoral immune cells directly contribute to the viability of the growing tumor (114). Techniques that incorporate spatial information also offer the ability to determine direct cell-cell interaction using Cell Neighborhood Analysis (204) and predict the roles of immune cells (205). These functional states can serve as additional prognostication metrics, as several studies to date have already defined the presence of bulk TAMs and MDSCs in tumor parenchyma vs. stroma in terms of patient outcomes (19, 40, 100, 206, 207). Further work in associating the added dimension of space to the tumor immune microenvironment is required to fully understand the complex interplay between myeloid cells and malignancies.

## Author Contributions

VD, GJ, SM, S-HC, and P-YP contributed to the intellectual content of the manuscript and contributed to the drafting of the manuscript. All authors read and approved of the final manuscript.

## Conflict of Interest

The authors declare that the research was conducted in the absence of any commercial or financial relationships that could be construed as a potential conflict of interest.

## Abbreviations

TIME: tumor-immune microenvironment
TAM: tumor-associated macrophage
MDSC: myeloid-derived suppressor cell
M-MDSC: monocytic myeloid-derived suppressor cell
scRNAseq: single-cell RNA sequencing
TRM: tissue resident macrophages
TLR: toll-like receptor
IFN: interferon
GM-CSF: granulocyte-macrophage colony-stimulating factor
STAT: signal transducer and activator of transcription
C-EBP: CCAAT/enhancer-binding protein
IRF: interferon regulatory factor
ROR: retinoic acid-related orphan receptor
CCL: C-C motif chemokine ligand
M-CSF/CSF1: colony stimulating factor 1
VEGF: vascular endothelial growth factor
IL: interleukin
PD-1: programmed cell death 1
PD-L1: programmed cell death ligand 1
CTLA: cytotoxic lymphocyte antigen
iNOS: inducible nitric oxide synthase
PPAR: peroxisome proliferator activated receptor
SOCS: suppressor of cytokine signaling
ARG1: arginase 1
IDO: indoleamine-pyrrole 2,3-dioxygenase
Bcl-xL: B-cell Lymphoma-extra large
ROS: reactive oxygen species
GCN2: general control non-derepressible 2
CREB: CAMP response element-binding protein
ATF: activating transcription factor
TGF: transforming growth factor
SPARC: secreted protein acidic and rich in cysteine, osteonectin
CCR: C-C motif chemokine receptor
MARCO: macrophage receptor with collagenous structure
NRP2: neuropilin 2
APOE: apolipoprotein E
IFITM1: interferon-induced transmembrane protein
TSPO: translocator protein
NSCLC: non-small-cell lung cancer
LILRB: leukocyte immunoglobulin like receptor B
PIRB: paired immunoglobulin-like receptor B
IL1RN: interleukin 1 receptor antagonist
NFKBIA: nuclear factor κ B inhibitor α, I κ B α
VISTA: V-domain Ig suppressor of T cell activation
CNS: central nervous system
GBM: glioblastoma multiforme
HIF: hypoxia-induced factor
PDAC: pancreatic ductal adenocarcinoma
TME: tumor microenvironment
PMN-MDSC: polymorphonuclear myeloid-derived suppressor cell
α-: anti-.
