# Corrigendum: Analyzing One Cell at a TIME: Analysis of Myeloid Cell Contributions in the Tumor Immune Microenvironment

**DOI:** 10.3389/fimmu.2020.645213

**Published:** 2021-02-01

**Authors:** Vitaliy Davidov, Garrett Jensen, Sunny Mai, Shu-Hsia Chen, Ping-Ying Pan

**Affiliations:** ^1^ Texas A&M College of Medicine, Bryan, TX, United States; ^2^ Center for Immunotherapy Research, Cancer Center of Excellence, Houston Methodist Research Institute, Houston, TX, United States

**Keywords:** MDSC, TAM, TIME, sc-RNAseq, reprogram

In the original article, we neglected to include the funder Texas A&M Open Access to Knowledge Fund (OAKFund), supported by the University Libraries, to VD; NIH R01 CA127483, R01 CA204191, R01 CA208703 to S-HC; and NIH R01 CA188610 to P-YP. S-HC is the Emily Hermann Chair in Immunology Research and this work was supported in part by that endowment.

The authors apologize for this error and state that this does not change the scientific conclusions of the article in any way. The original article has been updated.

